# Low serum lymphocyte level is associated with poor exercise capacity and quality of life in chronic obstructive pulmonary disease

**DOI:** 10.1038/s41598-020-68670-3

**Published:** 2020-07-16

**Authors:** Sung Woo Moon, Ah Young Leem, Young Sam Kim, Ji-Hyun Lee, Tae-Hyung Kim, Yeon-Mok Oh, Hyejung Shin, Joon Chang, Ji Ye Jung, KOLD Study Group

**Affiliations:** 1 Division of Pulmonology, Department of Internal Medicine, Severance Hospital, Yonsei University College of Medicine, 50-1 Yonsei-ro, Seodaemun-gu, Seoul, 03722 Republic of Korea; 2 Division of Pulmonology and Allergy, Department of Internal Medicine, CHA Bundang Medical Center, CHA University, 59 Yatap-ro, Seongnam, 13496 Republic of Korea; 3 Division of Pulmonary and Critical Care Medicine, Department of Internal Medicine, Hanyang University Guri Hospital, Hanyang University College of Medicine, 153 Gyeongchun-ro, Guri, 11923 Republic of Korea; 4 Department of Pulmonary and Critical Care Medicine and Clinical Research Center for Chronic Obstructive Airway Diseases, Asan Medical Center, University of Ulsan College of Medicine, 88 Olympic-ro 43-gil, Songpa-gu, Seoul, 05505 Republic of Korea; 5 Biostatistics Collaboration Unit, Department of Biomedical Systems Informatics, Yonsei University College of Medicine, 50-1 Yonsei-ro, Seodaemun-gu, Seoul, 03722 Republic of Korea

**Keywords:** Chronic obstructive pulmonary disease, Predictive markers

## Abstract

The purpose of this study was to evaluate the association of serum lymphocyte level with several clinical parameters in COPD. The study population included 451 COPD patients from the Korean Obstructive Lung Disease cohort study. Serum lymphocyte level was measured every year along with various clinical parameters, such as lung function, 6-min walking (6 MW) distance, quality of life using COPD assessment test (CAT) and St. George's Respiratory Questionnaire (SGRQ) scores, exacerbations, and survival. Serum lymphocyte level less than 20% was considered as a low lymphocyte level. Normal lymphocyte and low lymphocyte groups comprised of 409 (90.7%) and 42 (9.3%) patients, respectively. Clustered analysis showed that patients in low lymphocyte group had a lower post-bronchodilator forced expiratory volume in 1 s % predicted (estimated mean =  − 5.70%; *P* = 0.001), a lower forced vital capacity % predicted (estimated mean =  − 5.63%; *P* = 0.005), a shorter 6 MW distance (estimated mean =  − 41.31 m; *P* < 0.001), a higher CAT score (estimated mean = 2.62; *P* = 0.013), and a higher SGRQ score (estimated mean = 10.10; *P* < 0.001). Serum lymphocyte level was not associated with frequent acute exacerbations nor mortality. Low serum lymphocyte group showed poorer pulmonary function, lower 6 MW distance, and worse quality of life. Serum lymphocyte levels could be a simple and widely available predictive marker for variable clinical outcomes in COPD patients.

## Introduction

Chronic obstructive pulmonary disease (COPD) is a chronic respiratory disease characterized by persistent airflow limitation and increased inflammatory responses^1,2^. In the pathogenesis of COPD, chronic inflammation of the airways and the lung parenchyma has a critical role resulting goblet cell proliferation, glandular hyperplasia, fibrosis, collapse of the small airways, and parenchymal destruction^3^. However, evidences are increasing that COPD also results in systemic inflammation and extra-pulmonary manifestations^4^.

In several studies, it has been suggested that patients with COPD have increased levels of serum inflammatory markers^5–7^. Up to 70% of patients with COPD have at least one elevated serum inflammatory parameter^6^. However, use of common plasma measures and biomarkers such as procalcitonin, C-reactive protein, and white blood cell count, in routine clinical practice has limitations because of their low reproducibility to predict the prognosis in patients with COPD^8^.

Although lymphocytes play a critical role in the pathogenesis of COPD involving airway and parenchymal inflammation, patients with COPD have also shown a decreased in lymphocyte counts^8–11^. Low lymphocyte count is one of the markers of stress response and the low ratio of lymphocyte in the peripheral blood have the potential to indicate the pathological condition of chronic inflammation^8,10^. A relatively low lymphocyte count is known to as a negative prognostic factor in patients with stable coronary heart disease, congestive heart failure, acute myocardial infarction, and the elderly population^12–15^.

However, the role of serum lymphocyte level has not been fully validated in patients with COPD. Prediction of risk for various clinical outcomes with this simple serum measurement should be useful in clinical practice^16^. This study aimed to investigate the association of repeatedly and simultaneously measured serum lymphocyte level with various clinical parameters including pulmonary function, exercise capacity, quality of life (QOL), exacerbation, and survival in a Korean COPD cohort study.

## Methods

### Study subjects

From the patients enrolled in the Korean Obstructive Lung Disease (KOLD) study, this study evaluated 451 patients with COPD. The KOLD cohort study enrolled patients over 18 years-of-age with COPD or asthma from 17 hospitals in South Korea between 2005 and 2015^17,18^. The inclusion criteria were as follows: post-bronchodilator ratio of forced expiratory volume in 1 s to forced vital capacity (FEV_1_/FVC) of < 0.7, > 40 years of age, smoking history of ≥ 10 pack-years, and no or minimal abnormality other than typical findings of COPD (such as bullous change, hyperinflation, emphysema, increased bronchovascular markings, etc.) on chest radiography^2,17,18^. Patients with respiratory diseases other than obstructive lung disease (e.g., previous pulmonary resection, tuberculosis-destroyed lung, and bronchiectasis) and those with a recent (8 weeks before screening) exacerbation or other respiratory illness (such as upper respiratory infection or pneumonia) were excluded^17,18^. However, patients who recovered from an exacerbation and had been stable for more than 8 weeks were included in the study. After an initial enrollment visit, the patients were followed up every 3 months and demographics data, pulmonary function test, St. George's Respiratory Questionnaire (SGRQ), and 6-min walking (6 MW), and laboratory test were collected or performed every year in a stable condition for a least 4 weeks^17,18^. Acute exacerbations were assessed monthly through telephone contact and questionnaires every 3 months during hospital visit^17,18^.

### Assessment tests

The percentage of lymphocytes was defined as: (total lymphocytes / total leukocytes) × 100. In our laboratories, the normal range of the lymphocyte percentage is 20 to 51%. Patients with a serum lymphocyte percentage < 20% were assigned to the low lymphocyte group and those with a serum lymphocyte percentage of 20–51% were assigned to the normal lymphocyte group.

The low and normal lymphocyte groups were compared using different assessment tools: pulmonary function tests, the 6-min walking (6 MW) distance, QOL as measured by the COPD Assessment Test (CAT) and the St. George’s Respiratory Questionnaire (SGRQ), exacerbations, and mortality. All assessments were repeated every year with a simultaneous measurement of the lymphocyte percentage. The 6 MW distance was measured on a 20-m walking course^19^. Exacerbation was defined as aggravation of one of three symptoms (dyspnea, cough, or sputum) for 2 or more days requiring an unscheduled hospital visit for additional treatment of systemic steroid or antibiotics, emergency room visit, or hospitalization. Frequent exacerbation was defined as having two or more exacerbations per year. Death and the cause of death were assessed during follow-up period.

### Statistical methods

Student’s *t* test was used to compare continuous variables, whereas Pearson’s χ^2^ test was used to compare categorical variables between the low lymphocyte and normal lymphocyte groups. Data were presented as means ± standard deviation and numbers (percentage).

We assessed the relationship of the serum lymphocyte percentage with lung function, the 6 MW distance, QOL, exacerbation, and the survival in patients with COPD. Both the serum lymphocyte percentage and clinical outcomes were measured at the same time every year. Two effects of the serum lymphocyte percentage on repeatedly measured outcomes were analyzed: the effect of baseline level and the effect of overall level. First, to assess how the baseline serum lymphocyte percentage affected repeatedly measured continuous longitudinal datasets of lung function, 6 MW distance, dyspnea, and QOL, we conducted analysis of linear mixed model (LMM)^20^. To assess how the baseline serum lymphocyte percentage affected dichotomous longitudinal datasets of exacerbations, we conducted analysis of generalized estimating equations^21^. Second, we conducted clustered analysis to assess how the overall level of serum lymphocyte percentage including the baseline level affected repeatedly measured clinical outcomes at the same time. In contrast to the first approach, we considered all repeated serum lymphocyte percentage measurements.

To investigate the relationship between serum lymphocyte percentage and mortality, multivariable Cox proportional hazard models were employed in which two analyses were also conducted: a Cox regression with the baseline level of serum lymphocyte percentage as a covariate and a time-dependent Cox regression with the repeatedly measured serum lymphocyte percentage as a covariate.

To assess the effect of serum lymphocyte on lung function, the LMM model was fitted with age, gender, BMI, and smoking status (former or current) as additional covariates. Age, gender, BMI, smoking status (former or current), and FEV_1_% predicted were included as covariates in the LMM models for 6 MW distance, QOL, and in the Cox regression model. An adjusted *P* value < 0.05 was considered to indicate significance. SPSS version 25 (SPSS, Chicago, IL, USA) and SAS software, version 9.4 (SAS Institute, Inc., Cary, NC, USA) were used for all analyses.

### Ethics approval and informed consent

This study was performed in accordance with the provisions of the Declaration of Helsinki. The study protocol was approved by the institutional review boards of the 17 hospitals included in the KOLD Cohort (Asan Medical Center, Hanyang University Guri Hospital, Inje University Ilsan Paik Hospital, Bundangcha Hospital, Kangbook Samsung Medical Center, Ewha Womans University Mokdong Hospital, Kangwon National University Hospital, Korea University Anam Hospital, Seoul National University Hospital, Seoul National University Bundang Hospital, Hallym University Medical Center, Konkuk University Medical Center, Ajou University Hospital, National Medical Center, The Catholic University of Korea Seoul St Mary’s Hospital, The Catholic University of Korea Yeouido St Mary’s Hospital, Severance Hospital).

### Consent for publication

All study participants provided informed consent.

## Results

### Baseline characteristics

The baseline characteristics of the patients are shown in Table 1. Among the 451 patients, 409 (90.7%) and 42 (9.3%) were categorized into normal and low lymphocyte groups, respectively. The mean age, sex ratio, and proportion of patients with smoking status were similar between the groups. BMI (23.3 kg/m^2^ vs. 21.6 kg/m^2^; *P* = 0.009), FEV_1_% predicted (55.7% vs. 47.1%; *P* = 0.001), FVC % predicted (91.0% vs. 84.9%; *P* = 0.030), and FEV_1_/FVC (47.7% vs. 41.6%; *P* = 0.001) were significantly higher in the normal lymphocyte group. The proportion of GOLD III–IV was higher in the low lymphocyte group. The 6 MW distance (428 m vs. 360 m; *P* < 0.001) was longer and the SGRQ score (31.2 vs. 41.9; *P* < 0.001) was lower in the normal lymphocyte group. Mean follow-up duration was 6.0 years. Mortality during the follow-up period was significantly higher in the low lymphocyte group than that in the normal lymphocyte group (28.6% vs. 12.0%; *P* = 0.006). During the follow-up period, 58 (12.9%) patients expired. The causes of deaths consist of respiratory diseases (37.9%), cancer (24.1%), cardiovascular diseases (15.5%), others (5.2%), and unknown causes (17.2%). The serum lymphocyte percentage over the years did not vary and is depicted in Supplementary Fig. 1.

**Table 1:** Baseline characteristics of the normal and low lymphocyte groups.

	Total (n = 451)	Normal lymphocyte^a^ (n = 409)	Low lymphocyte^b^ (n = 42)	*P* value
Sex, male	437 (96.9)	395 (96.6)	42 (100)	0.630
Age, years	67.5 ± 7.6	66.8 ± 7.3	66.6 ± 6.9	0.874
Body mass index, kg/m^2^	22.9 ± 3.2	23.3 ± 3.2	21.6 ± 3.6	0.009
Smoking status				0.401
Current smoker	157 (34.8)	145 (35.5)	12 (28.6)	
Former smoker	294 (65.2)	264 (64.5)	30 (71.4)	
Pulmonary function				
FEV_1_, L	1.65 ± 0.56	1.69 ± 0.55	1.32 ± 0.55	< 0.001
FEV_1_, % predicted	54.9 ± 16.2	55.7 ± 15.7	47.1 ± 19.1	0.001
FVC, L	3.50 ± 0.78	3.54 ± 0.78	3.12 ± 0.74	0.001
FVC, % predicted	90.4 ± 17.6	91.0 ± 17.2	84.9 ± 19.9	0.030
FEV_1_/FVC, %	47.1 ± 11.2	47.7 ± 11.0	41.6 ± 11.7	0.001
GOLD^c^				0.007
GOLD I	24 (5.3)	22 (5.4)	2 (4.8)	
GOLD II	249 (55.3)	234 (57.4)	15 (35.7)	
GOLD III	151 (33.6)	134 (32.8)	17 (40.5)	
GOLD IV	26 (5.8)	18 (4.4)	8 (19.0)	
6 MW distance, m	422 ± 88	428 ± 85	360 ± 97	< 0.001
CAT Score	13.8 ± 7.4	13.6 ± 7.3	15.8 ± 7.7	0.272
SGRQ score	32.2 ± 17.9	31.2 ± 17.5	41.9 ± 19.3	< 0.001
Number of exacerbation per year	0.48 ± 0.79	0.46 ± 0.78	0.67 ± 0.85	0.100
Follow-up duration, years	6.0 ± 3.9	6.1 ± 3.9	4.9 ± 3.1	0.052
Death during follow-up	58 (12.9)	46 (12.0)	12 (28.6)	0.006

Data are presented as numbers (percentages) or means ± standard deviation, unless otherwise indicated.

*FEV*_1_ forced expiratory volume in 1 s, *FVC* forced vital capacity, *GOLD* global initiative for chronic obstructive lung disease, *6 MW* 6-min walking, *CAT* COPD assessment test, *SGRQ* St. George's respiratory questionnaire.

^a^Normal lymphocyte group was defined as those with serum lymphocyte percentage 20 to 51%.

^b^Low lymphocyte group was defined as those with serum lymphocyte percentage < 20%.

^c^*P* value for comparison between COPD patients with GOLD I–II and those with GOLD III–IV.

### Pulmonary function, 6-min walking distance and quality of life

Table 2 shows the results of the regression analysis for the association of serum lymphocyte percentage with the pulmonary function, adjusted with age, sex, BMI, and smoking status. In the baseline analysis, a higher absolute serum lymphocyte percentage was related with a higher FEV_1_% predicted and a higher FEV_1_/FVC ratio, while these lung function parameters were similar between the normal and low lymphocyte groups. Clustered analysis showed that a higher absolute overall level of serum lymphocyte percentage was related with a higher FEV_1_% predicted (estimated mean = 0.25%; *P* = 0.002) and a higher FEV_1_/FVC ratio (estimated mean = 0.15%; *P* = 0.003). Low overall level lymphocyte group showed lower FEV_1_% predicted (estimated mean =  − 5.70%; *P* = 0.001) and lower FVC % predicted (estimated mean =  − 5.63%; *P* = 0.005) than those of the normal lymphocyte group.

**Table 2:** Multivariate regression analysis for the effect of serum lymphocyte % on pulmonary function.

	FEV_1_, %		FVC, %		FEV_1_/FVC, %	
	Estimated mean (SE)	*P* value	Estimated mean (SE)	*P* value	Estimated mean (SE)	*P* value
*Baseline lymphocyte*, %						
Lymphocyte, %	0.32 (0.09)	< 0.001	0.14 (0.09)	0.122	0.19 (0.06)	0.001
Lymphocyte groups						
Normal lymphocyte^a^	Reference		Reference		Reference	
Low lymphocyte^b^	− 8.51 (6.60)	0.198	− 12.42 (9.37)	0.185	− 1.18 (4.19)	0.778
*Clustered analysis*						
Lymphocyte, %	0.25 (0.08)	0.002	0.12 (0.09)	0.293	0.15 (0.05)	0.003
Lymphocyte groups						
Normal lymphocyte^a^	Reference		Reference		Reference	
Low lymphocyte^b^	− 5.70 (1.71)	0.001	− 5.63 (1.98)	0.005	− 1.99 (1.21)	0.100

Adjusted for age, gender, body mass index, and smoking status (current or former).

*FEV*_1_ forced expiratory volume in 1 s, *FVC* forced vital capacity, *SE* standard error.

^a^Normal lymphocyte group was defined as those with serum lymphocyte percentage 20 to 51%.

^b^Low lymphocyte group was defined as those with serum lymphocyte percentage < 20%.

Table 3 shows the results of the regression analysis for the association of serum lymphocyte percentage with the 6 MW distance and quality of life indexes, adjusted with age, sex, BMI, smoking status, and baseline FEV_1_. In the baseline analysis, a higher absolute percentage of serum lymphocytes was related with a longer 6 MW distance and lower CAT and SGRQ scores. In the clustered analysis, a higher overall absolute percentage of serum lymphocytes was related with a longer 6 MW distance and lower CAT and SGRQ scores. Similarly, in the between-group comparison, the low lymphocyte group showed a lower 6 MW distance (estimated mean =  − 41.31 m; *P* < 0.001), a higher CAT score (estimated mean = 2.62; *P* = 0.013), and a higher SGRQ score (estimated mean = 10.10; *P* < 0.001) in the clustered analysis.

**Table 3:** Multivariate regression analysis of the effect of serum lymphocyte % on 6-min walking distance and quality of life.

	6 MW distance, m		CAT score		SGRQ score	
	Estimated mean (SE)	*P* value	Estimated mean (SE)	*P* value	Estimated mean (SE)	*P* value
*Baseline lymphocyte*, %						
Lymphocyte, %	1.56 (0.45)	0.001	− 0.16 (0.04)	< 0.001	− 0.37 (0.10)	< 0.001
Lymphocyte groups						
Normal lymphocyte^a^	Reference		Reference		Reference	
Low lymphocyte^b^	− 32.26 (64.67)	0.618	− 1.37 (6.00)	0.820	− 1.53 (11.15)	0.891
*Clustered analysis*						
Lymphocyte, %	1.88 (0.39)	< 0.001	− 0.16 (0.04)	< 0.001	− 0.42 (0.09)	< 0.001
Lymphocyte groups						
Normal lymphocyte^a^	Reference		Reference		Reference	
Low lymphocyte^b^	− 41.31 (8.94)	< 0.001	2.62 (1.05)	0.013	10.10 (2.02)	< 0.001

Adjusted for age, sex, body mass index, smoking status (current or former), and baseline forced expiratory volume in 1 s, % predicted.

*SE* standard error, *6 *MW 6-min walking, *CAT* COPD assessment test, *SGRQ* St. George's respiratory questionnaire.

^a^Normal lymphocyte group was defined as those with serum lymphocyte percentage 20 to 51%.

^b^Low lymphocyte group was defined as those with serum lymphocyte percentage < 20%.

### Frequent exacerbation and mortality

The serum lymphocyte percentage was not associated with frequent exacerbations after adjustment for age, sex, BMI, smoking status, and baseline FEV_1_ (Table 4).

**Table 4:** Multivariate regression analysis of the effect of serum lymphocyte % on frequent exacerbation.

	Odds ratio (95% CI)	*P* value
*Baseline lymphocyte*, %		
Lymphocyte, %	0.98 (0.95–1.00)	0.056
Lymphocyte groups		
Normal lymphocyte^a^	Reference	
Low lymphocyte^b^	1.27 (0.71–2.29)	0.417
*Clustered analysis*		
Lymphocyte, %	0.99 (0.97–1.01)	0.175
Lymphocyte groups		
Normal lymphocyte^a^	Reference	
Low lymphocyte^b^	1.00 (0.63–1.59)	0.989

Adjusted for age, sex, body mass index, smoking status (current or former), and baseline forced expiratory volume in 1 s, % predicted. Frequent exacerbation was defined as having two or more exacerbations per year.

*CI* confidence interval.

^a^Normal lymphocyte group was defined as those with serum lymphocyte percentage 20 to 51%.

^b^Low lymphocyte group was defined as those with serum lymphocyte percentage < 20%.

Table 5 shows the results of the two analyses with multivariable Cox proportional hazard models: The Cox regression with the baseline level of serum lymphocyte percentage as a covariate and the time-dependent Cox regression with repeatedly measured serum lymphocyte percentage as a covariate. The results are adjusted for age, sex, smoking status, BMI, and baseline FEV_1_. No significant relationship was observed.

**Table 5:** Multivariable Cox’s proportional hazard analyses for relationship between serum lymphocyte % and mortality.

	Hazards ratio (95% CI)	*P* value
*Baseline lymphocyte*, %		
Lymphocyte, %	0.99 (0.96–1.03)	0.749
Lymphocyte groups		
Normal lymphocyte^a^	Ref	
Low lymphocyte^b^	1.27 (0.66–2.43)	0.478
*Overall level of lymphocyte*, %		
Lymphocyte, %	0.99 (0.95–1.05)	0.937
Lymphocyte groups		
Normal lymphocyte^a^	Ref	
Low lymphocyte^b^	1.92 (0.77–4.80)	0.163

Adjusted for age, sex, body mass index, smoking status (current or former), and baseline forced expiratory volume in 1 s, % predicted.

*CI* confidence interval.

^a^Normal lymphocyte group was defined as those with serum lymphocyte percentage 20 to 51%.

^b^Low lymphocyte group was defined as those with serum lymphocyte percentage < 20%.

## Discussion

We investigated the serum lymphocyte percentage in Korean patients with COPD and its relationship with several clinical parameters of lung function, exercise capacity, QOL, exacerbation, and mortality. The major strength of this study is that it was a long-term multicenter study, which enhances the statistical reliability of the results. The present analysis showed that a low serum lymphocyte percentage is related with a lower lung function, a shorter 6 MW distance, and worse quality of life, as shown by increased CAT and SGRQ scores.

The 6 MW distance is a simple and inexpensive test that provides a global and integrated response of both physical (pulmonary and nonpulmonary factors) and psychological factors in patients with COPD^22^. The QOL is a composite concept that reflects the perception of an individual’s position within the living context^23^. It is an important domain for measuring the impact of chronic diseases^24^. Although acute exacerbation and the survival were not related with the serum lymphocyte percentage, the clinical significance of serum lymphocyte percentage in patients with COPD cannot be underestimated.

The present findings are in agreement with those of previous studies that attempted to use serum lymphocyte levels as a predictor in patients with COPD. Neutrophil to lymphocyte ratio (NLR) has shown to be an independent predictor of the prognosis in patients with COPD^25–28^. However, the reason NLR is related with the prognosis and whether lymphocytes in particular are independently associated with the prognosis in patients with COPD remained unclear. Acanfora et al. had shown that in elderly patients with COPD, decreased serum lymphocyte levels were related with increased 3-year mortality^8^.

Comparing our study with the previous study by Acanfora et al.^8^, the mortality did not rise as the serum lymphocyte percentage decreased in our study. In the study of Acanfora et al., patients were aged 65 years or older and had severe or very severe air flow limitation, suggesting a more severe COPD status than that of patients in our study. Furthermore, our study analyzed a longer follow-up period of mean 6 years for mortality. The proportion of death during the total follow-up period was significantly higher in the low lymphocyte group than that in the high lymphocyte group in our study as well.

The prognostic value of the lymphocyte count has been evaluated in cardiovascular diseases^29–32^. A low lymphocyte count was related with an increased risk for myocardial infarction and death in patients with acute chest pain, a non-diagnostic electrocardiogram, and normal troponin levels. In the English general population without pre-existing cardiovascular disease, low lymphocyte counts were associated with an increased short-term incidence of heart failure and coronary death^31^. The authors described the relationship of lymphopenia and cardiac events as a result of systemic stress and inflammatory activation, driven by tissue ischemia and oxidative stress.

It has been long known that chronic inflammation is a key pathogenic element in COPD and that lymphocytes play a crucial role in the pathogenesis of COPD^9,32^. In the histopathologic study, the bronchioles are obstructed by fibrosis and infiltration with macrophages and T lymphocytes^1^. In severe COPD, bronchoalveolar lavage or induced sputum shows a more marked increase in macrophages and neutrophils^1^, which may result in decreased relative percentage of lymphocytes. Moreover, lymphopenia may be a sign of an impaired immune system, increasing the risk of infection, although lymphopenia was not associated with COPD exacerbation in our study where moderate or severe exacerbation were evaluated. However, lymphocytes would be a comprehensive indicator of the general health status as a low lymphocyte count was related with heart failure, chronic kidney disease, atrial fibrillation, and COPD in patients admitted for acute heart failure^27^. Elderly adults have a lower lymphocyte level than that of young adults and apoptosis is associated with cellular depletion^33^. However, lymphopenia would be the result of a systemic response to psychological and physiological stress. Increased production of cortisol, catecholamines, and proinflammatory cytokines may decrease the production of lymphocytes, increase the apoptosis, or make redistribution of lymphocytes^30,34^.

Another reason for our results may be that a low lymphocyte level is also related with malnutrition. The relationship between malnutrition and lymphocytopenia is well known^35^, and lymphocytes are included in the prognostic nutritional index, a nutritional marker. Patients with stable COPD generally have a normal lymphocyte count, but lymphopenia occurs during acute weight loss in the setting of severe illness or acute respiratory failure. Refeeding and weight gain in patients with COPD with recent weight loss in a state of mild malnutrition were associated with a significant increase in the lymphocyte count^36^. BMI is another essential marker for assessment of the nutritional status in COPD; however, a low lymphocyte level was significantly associated with worse exercise capacity and quality of life even after adjustment with BMI in this analysis^37^.

Our study has several limitations. First, the proportion of patients in the low lymphocyte group was relatively small compared to that of patients in the normal lymphocyte group. However, the repeated measurements over a long-term period are the strength of this study. Second, KOLD cohort recruited COPD patients with smoking history, which led to male dominance in the study population. In Korea, smoking rate of women is low at 5–6%^38^. Therefore, this study may not represent the general pool of patients with COPD. Smoking is known to reduce the serum lymphocyte level^39^. Therefore, current or former smoking status was included as a co-variate in the analysis. Third, other conditions that might influence the serum lymphocyte level were not included in the analysis, such as a history of infection or medication. Fourth, lymphocyte sub-types, such as B cells and T cells, were not further investigated; thus, the respective prognostic role of deficits of the cellular and humoral immunity could not be assessed.

In conclusion, this COPD cohort study included simultaneously repeated measurement of serum lymphocyte percentage and clinical consequences every year.

Our study showed that a lower percentage of lymphocytes in the serum is related with several negative clinical outcomes in COPD. In patients with a low serum lymphocyte level, the pulmonary function was lower, the 6 MW distance was shorter, and the QOL was worse. Serum lymphocyte percentage could be a simple, intuitive, reproducible, widely available, and inexpensive predictive marker for variable clinical outcomes in patients with COPD.

## Supplementary information

Supplementary file1 (PDF 480 kb)

## Data Availability

The datasets used and/or analyzed are available from corresponding author upon reasonable request.
