# Supplementary material for: Low serum lymphocyte level is associated with poor exercise capacity and quality of life in chronic obstructive pulmonary disease
Source: Sci Rep. 2020 Jul 16;10:11700. doi: 10.1038/s41598-020-68670-3 (PMC7366616; doi:10.1038/s41598-020-68670-3)

# Supplementary Materials

**TITLE: Low serum lymphocyte level is associated with poor exercise capacity and quality of life in chronic obstructive pulmonary disease**

Sung Woo Moon[1], Ah Young Leem[1], Young Sam Kim[1], Ji-Hyun Lee[2], Tae-Hyung Kim[3], Yeon-Mok Oh[4], Hyejung Shin[5], Joon Chang[1], Ji Ye Jung[1], and KOLD Study Group

**Affiliations:**

[1]Division of Pulmonology, Department of Internal Medicine, Severance Hospital, Yonsei University College of Medicine., Seoul, Republic of Korea

[2]Department of Internal Medicine, CHA Bundang Medical Center, CHA University, Seongnam, Republic of Korea

[3]Division of Pulmonary and Critical Care Medicine, Hanyang University Guri Hospital, Hanyang University College of Medicine, Guri, Republic of Korea

[4]Department of Pulmonary and Critical Care Medicine and Clinical Research Center for Chronic Obstructive Airway Diseases, Asan Medical Center, University of Ulsan College of Medicine, Seoul, Republic of Korea

[5]Biostatistics Collaboration Unit, Department of Biomedical Systems Informatics, Yonsei University College of Medicine, Seoul, Korea

Supplementary Figure 1. Serum lymphocyte percent (%) over the follow-up period.

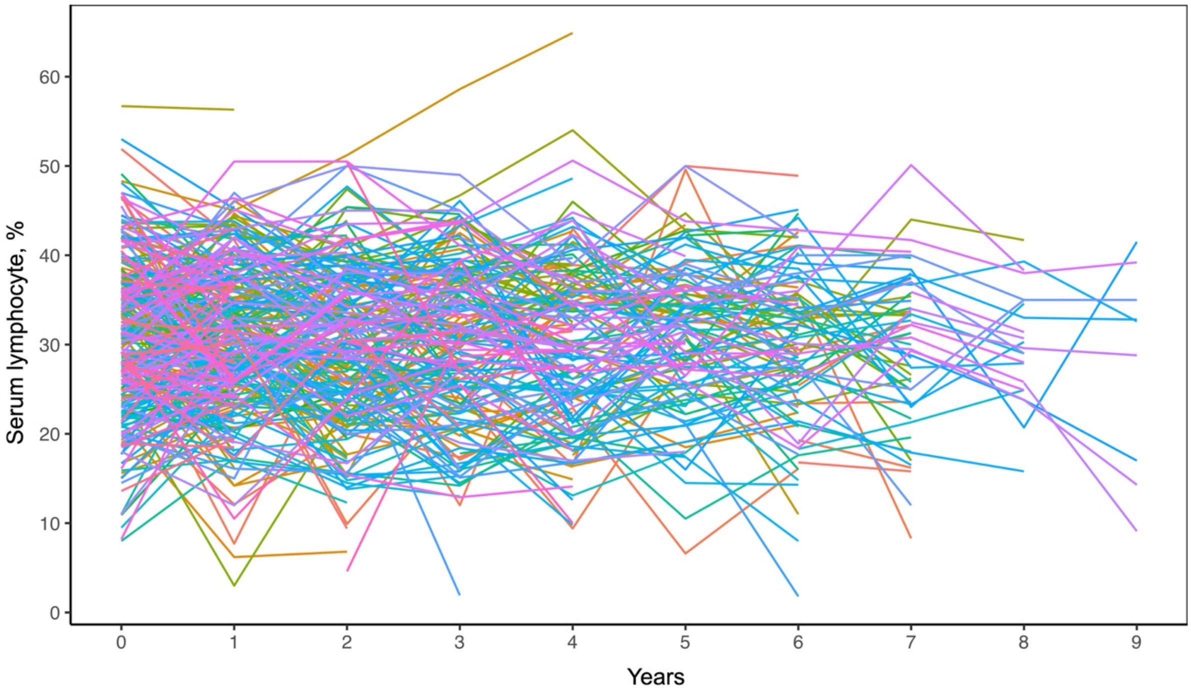

Supplement: Supplementary file 1 — Supplementary file1 (PDF 480 kb) [file 41598_2020_68670_MOESM1_ESM.pdf]
